# The many faces of p97/Cdc48 in mitochondrial homeostasis

**DOI:** 10.1042/EBC20253045

**Published:** 2026-01-07

**Authors:** Jonathan Ram, Michael H Glickman

**Affiliations:** 1 Faculty of Biology, Technion-IIT, Haifa, 32000, Israel

**Keywords:** Cdc48, ERAD, MAD, mitochondria, mitostasis, P97, proteasome, ubiquitin, VCP

## Abstract

Through its various roles in protein quality control, membrane dynamics, and cellular survival pathways, the AAA+ ATPase p97/valosin-containing protein emerges as a significant regulator of mitochondrial homeosta sis. This review comprehensively examines the multifaceted functions of p97 in mitochondrial biology, spanning from mitochondria-associated degradation to newly discovered functions in organellar cross-talk and disease pathogenesis. Underlying its cellular importance, p97 mutations are found in amyotrophic lateral sclerosis and frontotemporal dementia. To elucidate its mechanistic contribution to these processes, we provide a detailed table (Table 1) listing all known mitochondrial Cdc48/p97 substrates and associ ated proteins, categorized by their respective pathways. Recruitment to most of these substrates occurs by specialized adaptors, including Doa1/phospholipase A-2-activating protein, UBXD8, and UBXN1. p97 orchestrates the extraction and proteasomal degradation of outer mitochondrial membrane proteins, which are essential for maintaining mitochondrial integrity. For example, by controlling the turnover of fusion factors MFN1/2 and fission machinery, p97 regulates mitochondrial dynamics. p97 also governs apoptotic signaling through the regulated degradation of anti-apoptotic factors, such as myeloid cell leukemia-1 and VDAC, thereby modulating mitochondrial permeability. In mitophagy, p97 enables the clearance of damaged organelles by extracting ubiquitinated substrates and recruiting autophagy machinery. Beyond proteolysis, p97 facilitates recycling of endoplasmic reticulum-mitochondria contact sites through regulation of UBXD8-dependent lipid metabolism. Recent discoveries have revealed p97’s involvement in pathogen host interactions and circular RNA-mediated regulation, thereby expanding our understanding of its cellular functions. The emerging picture positions p97 as an integrative hub co-ordinating mitochondrial protein homeostasis, organellar dynamics, and cell fate decisions, with therapeutic potential for metabolic and neurodegenerative disorders.

## Introduction

The proteome of eukaryotic cells undergoes constant turnover through cycles of synthesis and degradation to maintain homeostasis. Protein degradation is governed by the ubiquitin-proteasome system (UPS), which consists of over 1000 different proteins. Ubiquitination is a three-part process, starting with activation of ubiquitin (Ub) by the E1 enzyme, followed by transfer to an E2 conjugase, and finalized by conjugation to a substrate protein catalyzed by one of many substrate-specific E3 ligases [1]. Ubiquitination was initially discovered in the context of protein degradation by the proteasome but was later found to be involved also in protein trafficking and myriad signaling pathways. The homo-hexamer AAA+ATPase p97/valosin-containing protein (VCP) (Cdc48 in yeast) is a widely spread auxiliary factor of the UPS for selecting, preparing, and targeting ubiquitinated substrates to the proteasome [2]. In the secretory pathway, faulty proteins are degraded by the proteasome in a process termed endoplasmic reticulum-associated degradation (ERAD). p97 is key to ERAD by extracting proteins from the ER and unfolding them through its internal catalytic channel before handing them over to the cytosolic proteasome for proteolysis. Since its discovery, ERAD has been thoroughly characterized and can be reviewed in detail elsewhere (3). p97 is also involved in other processes, such as the degradation of general proteasome substrates lacking an unfolded terminal region [3]. p97 is also involved in the extraction of subunits from complexes and unfolded proteins from aggregates [4,5]. To carry out these functions, adaptor proteins, such as NPL4 and UFD1, facilitate Ub binding to p97 and substrate insertion into its central pore. Over 30 such adaptors are known in mammals, affecting p97 localization, substrate specificity, and enzymatic kinetics [6,7]. In this review, we will focus on a subset of these adaptors, UBXD1, UBXD8, UBXN1, and phospholipase A-2-activating protein (PLAA), which are involved in mitochondrial proteostasis (Table 1).

Reversible and non-reversible inhibitors of p97 are useful tools available for research purposes, and some have entered clinical trials with the hope of breaking the dependency of cancerous cells on p97 [8]. By contrast, mutations in p97 are associated with amyotrophic lateral sclerosis (ALS) and frontotemporal dementia (FTD), highlighting its role in maintaining neuronal health [9,10]. These neurodegenerative diseases involve mitochondrial dysfunction, providing a link, whether direct or indirect, between p97 and mitochondria.

Mitochondria are a hub for cellular processes, including various metabolic pathways and the initiation of apoptosis. Mitochondria contain an outer mitochondrial membrane (OMM) and an inner mitochondrial membrane (IMM), with intermembrane space (IMS) between them and a luminal matrix. In intact mitochondria, only OMM proteins are exposed to the cytosol and UPS components, rendering the inner compartments inaccessible to the UPS. The vast majority of mitochondrial proteins, particularly at intra-mitochondrial compartments, are imported from the cytosol via narrow channels in the OMM. Continuous protein import is crucial for maintaining mitochondrial health. Defects in import threaten not only mitochondria but the entire cell, as they lead to the accumulation of non-imported precursors and proteotoxic stress [11]. Mitochondria form intricate tubular structures throughout the cell (rather than the textbook bean-shaped image), constantly undergoing fission and fusion events as part of mitochondrial homeostasis. Mitochondria and the ER also form connections and contact sites [endoplasmic reticulum-mitochondria contact sites (ERMCSs)] to facilitate lipid transfer, calcium signaling, and proteostasis regulation [12,13].

Due to oxidative phosphorylation carried out along the IMM, mitochondria generate reactive oxygen species (ROS) and other molecular radicals that can damage proteins, lipids, and DNA in—and in the vicinity of—mitochondria. Damaged mitochondria are surveyed by cytosolic quality control mechanisms and degraded in lysosomes through a process termed mitochondrial autophagy, or mitophagy for short. Mitophagy can occur via two distinct major pathways: PTEN-induced putative kinase 1 (PINK1)/Parkin-dependent (also known as Ub-dependent) and receptor-mediated (also known as Ub-independent), depending on the cause of mitophagy [14,15]. P97 plays a role in mitophagy. Here, we review the role of p97/VCP/Cdc48 in all aspects of mitochondrial biology.

## p97 in MAD

### MAD **of** OMM **proteins**

Degradation of membrane proteins at the ER depends on p97 activity; p97 is recruited to ubiquitinated proteins and extracts them from membranes to the cytosol, where they are later degraded in the 26S proteasome [16]. At the plasma membrane, ubiquitinated proteins are degraded in lysosomes via endocytosis, a process that is not directly dependent on p97. But what about OMM proteins? How are they extracted and degraded, and is p97 involved in this process?

**Figure 1:**
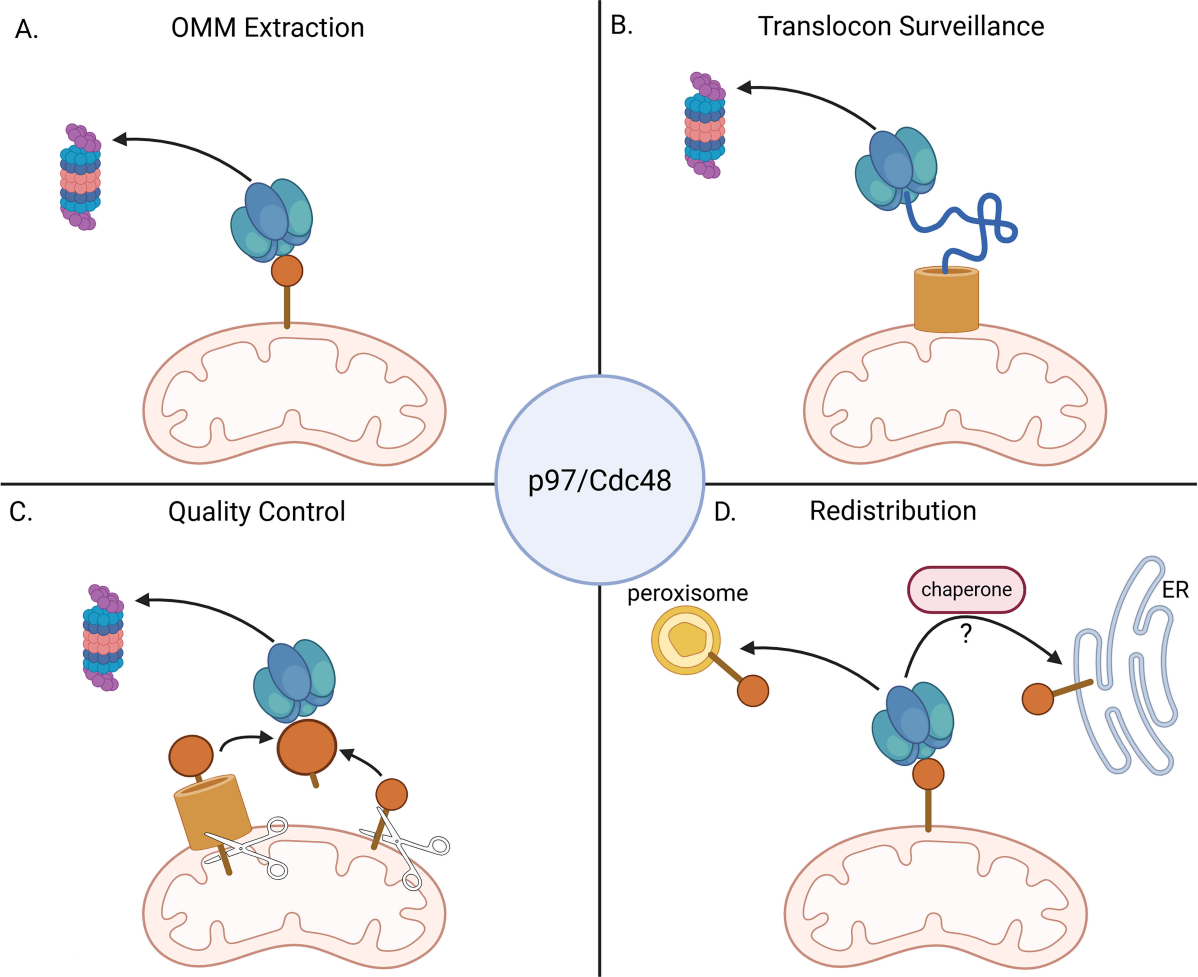
Potential mechanisms of p97 in mitochondrial protein homeostasis. **(A-B**) Mitochondria-associated degradation (MAD) of proteins from the OMM (**A**) or the TOM complex (**B**). **C**) Protein quality control of mitochondria-related proteins in the cytosol. These can be import-destined proteins or released fragments. **D**) Redistribution of mitochondrial proteins to other organelles. Created in BioRender. Lab, G. (2025) https://BioRender.com/0fm2clf.

**Figure 2:**
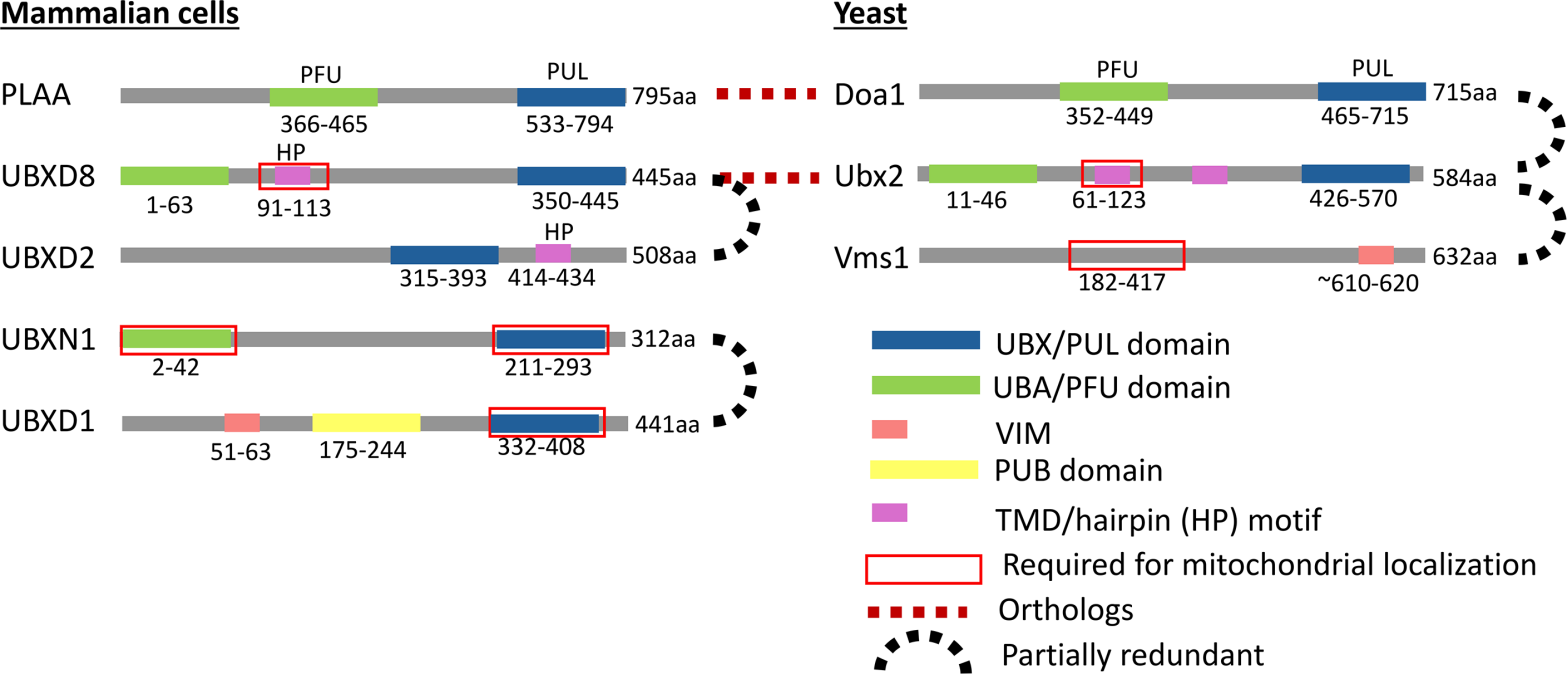
Functional domain organization of p97 adaptors in mitochondrial homeostasis. Schematic representation of yeast and mammalian p97/Cdc48 adaptors showing their functional domains, redundancy, and orthology. PUB, domain that binds the p97 C-terminal PIM motif; TMD, transmembrane domain; UBA/PFU, domains that mediate ubiquitin binding; UBX/PUL, domains that facilitate interaction with p97/Cdc48; VIM, VCP-interacting motif.

**Figure 3:**
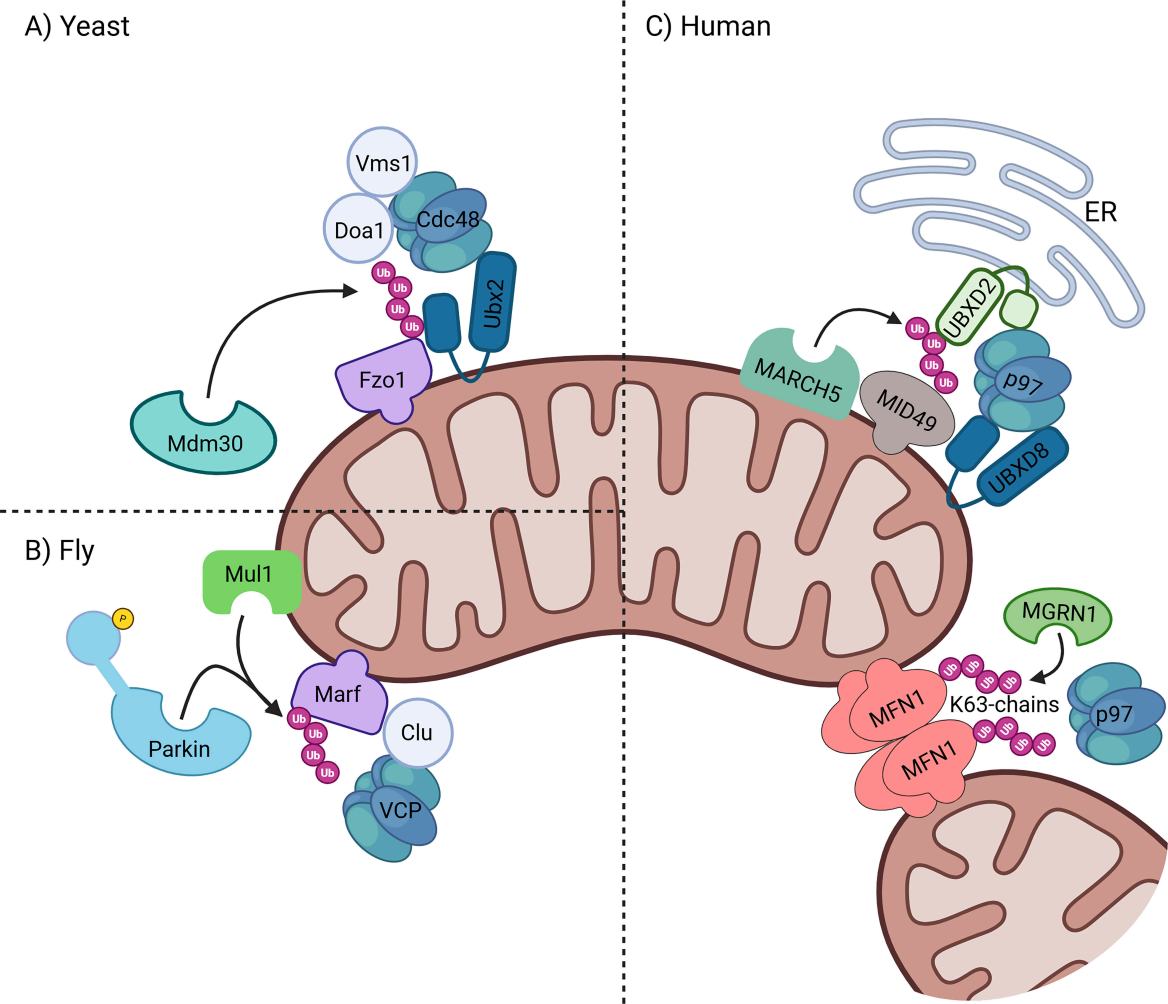
p97 in mitochondrial fusion and fission. (**A**) In yeast (*S. cerevisiae*), the mitofusin Fzo1 is ubiquitinated by Mdm30, recognized by Cdc48 and one or more of its cofactors (Ubx2, Doa1 or Vms1), extracted from the OMM and degraded in proteasomes B) in fly (*D. melanogaster*), the mitofusin Marf is ubiquitinated by Parkin or mul1, extracted by VCP with the aid of Clu and degraded in proteasomes. (**C**) in humans (*H. sapiens*), the fission factor MID49 is ubiquitinated by MARHC5, extracted by p97 and its cofactors (UBXD8 and UBXD2), and degraded by the proteasome. Following mitochondrial fusion, MFN1 complexes are ubiquitinated by MGRN1, disassembled and extracted by p97, and then degraded by proteasomes. Created in BioRender. Lab, G. (2025) https://BioRender.com/1w8dtb3.

The first footsteps in the discovery of the potential involvement of p97 in mitochondria-associated degradation (MAD) began with work on the yeast *S. cerevisiae*, where specific OMM proteins were stabilized by manipulating Cdc48 activity [17]. The first evidence for Ub-dependent proteasomal degradation of a mitochondrially anchored protein is that of the yeast mitofusin Fzo1 [18]. This example was predicted to serve as a prototype for a broader phenomenon and was nicknamed MAD for mitochondria-associated degradation, akin to the earlier discovered ERAD [19]. The process was shown to depend on the E3 ligase component, Mdm30, for ubiquitination and proteasomal degradation [18]. The question then became: “How is an OMM protein degraded by the cytosolic 26S proteasome?” (Figure 1A). The answer arrived soon, as it was found that Vms1 interacts with—and recruits—Cdc48 to mitochondria, facilitating the degradation of Fzo1 (Figure 2) [17]. A later report found that Vms1 translocated to mitochondria in response to oxidative stress via direct binding to OMM ergosterol peroxide, an oxidized sterol [20]. Additional evidence linking Cdc48 to Fzo1 turnover came from Arf1-11 mutant cells that display clustered Fzo1 and an aberrant mitochondrial phenotype. This phenotype can be reversed by the overexpression of Cdc48, leading to the degradation of Fzo1 and the restoration of normal mitochondrial morphology [21]. The mechanistic contribution of MAD, in this case, was proposed to serve a regulatory role: because Fzo1 is a key component of the fusion machinery, its degradation results in a decrease in the amount of Fzo1 available to mediate mitochondria fusion events (Figure 3A). By employing Ub-dependent degradation, the cell can carefully fine-tune the levels of mitofusins and fission factors to balance mitochondrial network dynamics and maintain healthy mitochondrial respiration.

In a key development that expanded the role of Cdc48 in MAD, a genetic screen aimed at identifying MAD regulators identified the Cdc48 adaptor Ub-binding protein, Doa1 [22], as crucial for the degradation of multiple OMM proteins [23]. Among these proteins are the first discovered MAD substrate Fzo1 (Table 1), the Ub E2 Mdm34, the AAA^+^ ATPase Msp1, and the translocase of the outer membrane (TOM) receptor Tom70. Degradation of the former three requires Npl4 and Ufd1 along with Doa1. Doa1 binds to polyubiquitinated substrates via its WD40 and PFU domains, with substrate binding preceded by Cdc48 binding through its PUL domain (Figure 2). In cells lacking *DOA1,* ubiquitinated Fzo1 and Mdm34 accumulate at the OMM, indicating a role for Cdc48-mediated extraction from the OMM.

In *A. thaliana,* CDC48A was proposed to extract the tail-anchored TOM receptor, TOM20, from the OMM in response to heat shock-induced polyubiquitination [24]. Following extraction, a shuttling factor called TTOP transports the extracted TOM protein to the proteasome for degradation. The E3 ligase involved in this process, as well as the CDC48 adaptors involved, remains unknown.

Beyond the selective and targeted degradation of OMM proteins, MAD can also participate in protein quality control of damaged or misfolded OMM proteins. For example, the products of two temperature-sensitive mutants, *sen2* and *sam35*, are ubiquitinated by different E3 ligases, Ubr1 and San1, respectively, leading to their extraction from the OMM by Cdc48-Npl4-Ufd1 and Ubx2 for proteasomal degradation [25]. This degradation also depends on the chaperone Hsp70/Ssa1 and its co-chaperone Hsp40/Sis1, as depletion of either slows the degradation rate.

### MAD of intramitochondrial proteins

MAD is not limited to OMM proteins, as it was later suggested to target even some inner mitochondrial compartments. An increasing number of intramitochondrial proteins have been found to be ubiquitinated, rendering them at least potentially as substrates for p97 [26]. Under oxidative stress induced by paraquat, Doa1 was shown to aid in the degradation of intramitochondrial proteins. A proteomic experiment performed in WT and Doa1-deficient cells, where ubiquitinated proteins were enriched from crude mitochondria, identified many potential MAD substrates enriched in Doa1-deleted cells [27]. Two potential substrates, Kgd1 and Pim1p, were further validated by other biochemical methods. The authors suggested a model in which Cdc48-aided retro-translocation of matrix proteins enables their MAD, although a more logical explanation would be that these MAD substrates are unimported proteins stuck in TOM channels (Figure 1B). Subsequent work demonstrated that Cdc48 is recruited to blocked TOM complexes by Ubx2, a UBX-UBA domain-containing protein (Figure 2) initially identified as supporting ERAD [28]. The mitochondrial proteins stuck in the TOM channel are ubiquitinated by Rsp5 and extracted by Cdc48 in a Ubx2-dependent manner. To emphasize the role in the unclogging of TOM complexes, this pathway was named mitochondrial protein translocation-associated degradation (mitoTAD) [29].

The mammalian homologue of Ubx2, UBXD8/FAF2 (Figure 2), was also shown to localize to the TOM complex, but it was suggested not to be involved in the extraction/degradation of stalled precursor proteins from the TOM complex [13]. Carbonyl cyanide m-chlorophenylhydrazone (CCCP) treatment blocks protein import to mitochondria and leads to the accumulation of precursors. A cycloheximide chase experiment showed that these precursors were degraded at the same rate in WT and UBXD8 KO cells, suggesting that UBXD8 is not required for mitoTAD in mammalian mitochondria. Two complementary studies have found that the extraction of stalled protein import intermediates from the TOM complex in mammals is p97-independent [30,31]. Emulating previous work in yeast, a mitochondrial GFP-fusion protein was used to clog TOM complexes to study its degradation pathway [30]. Unlike yeast, which employ Cdc48 for the extraction of clogged proteins [28], mammalian cells rely on the mitochondrial protease OMA1 to cleave the clogger in the IMS and release the globular GFP fragment to the cytosol. Another attempt to adapt the ‘clogger’ approach to mammalian cells used a fusion of an N-terminal fragment of MIC60 and the globular domain of DHFR [31]. Cells expressing this clogger can be treated with methotrexate, which binds tightly to DHFR, thereby blocking it from unfolding and preventing it from entering through the TOM channel. In this scenario too, p97 was unable to extract the mature clogger from TOM complexes. Ironically, as illustrated in Figure 1C, degradation of the cytosolic fragments of both ‘cloggers’ (GFP and DHFR) requires active p97 for proteasomal degradation [30,31], evoking the importance of Cdc48/p97 in the degradation of tightly folded proteasome substrates [3].

Evidently, the role of Cdc48 in the extraction of mitochondrial proteins from blocked TOM complexes is not conserved in mammalian cells, which appear to employ p97-independent strategies to confront this stress.

## p97 in membrane dynamics

The most studied role of p97 in mitochondrial membrane dynamics is its participation in the degradation of mammalian mitofusins. MFN1 and MFN2 drive mitochondrial fusion of the OMM in mammalian cells. p97 plays a role in the steady-state regulation of MFN1 levels for healthy mitochondria [32], and p97 inhibition generates elongated mitochondria in flies. Disassembly of MFN complexes and extraction of these large membrane-anchored GTPases by p97 promote their degradation by the proteasome, keeping levels of these mitofusins in check and thereby suppressing, delaying, or preventing fusion of the OMM [33]. If mitochondrial fission continues unabated, the result would be smaller, rounder, fragmented mitochondria. Moreover, 97-dependent disassembly of MFN2 complexes from ERMCS dissociates mitochondria from the ER by separating the ER-membrane-residing MFN2 from its OMM counterpart [33]. In addition to the obvious role of p97-aided degradation to limit fusion of mitochondria, p97 may also promote fusion by terminating the reaction. After tethering of mitochondria, the E3 ligase MGRN1 adds K63-linked chains to MFN1 complexes, which helps generate higher-order MFN1 oligomers (Figure 3C). Next, p97-dependent extraction and proteasomal degradation complete OMM fusion [34]. In this case, p97-dependent extraction is a prerequisite for mitochondrial fusion and maintenance of the enmeshed mitochondrial network under basal conditions. Overall, p97 regulates mitochondrial fusion by controlling MFN levels—preventing excessive elongation—while also promoting fusion completion through disassembly of MFN1 complexes.

Another role of P97 is regulation of mitochondrial fission factor levels. Following its ubiquitination by the E3 ligase MARCH5, degradation of the fission factor MID49 is mediated by two UBX-UBA proteins [35]. Mitochondria-localized UBXD8 (Figure 2) mitigates the p97-dependent degradation of MID49 in a cis manner*,* while ER-membrane-localized UBXD2 (Figure 2) does the same in a trans manner (Figure 3C). Moreover, mitochondria-localized UBXD8 enables the degradation of ER membrane proteins by recruiting p97 in a trans manner. These findings suggest that ER-membrane-residing and OMM-residing p97 adaptors are redundant (Figure 2) in the degradation of some ERMCS proteins.

A new avenue in p97 research recently arose with a pioneering study on p97’s indirect role in regulating membrane lipid dynamics [12]. Inhibition of p97, or depletion of either p97 or UBXD8, increased the amount of ERMCS in human embryonic Kidney 293 kidney 293 (HEK293) cells. Elevated ERMCS in UBXD8-depleted cells was not recapitulated by knockdown of other p97 adaptors, whether NPL4, UFD1, UBXD2, or UBXD7, establishing the unique role of UBXD8 in ERMCS. In this experiment, ERMCS membranes in UBXD8-deleted cells were enriched in saturated lipids. The involvement of UBXD8-p97 in lipid saturation was known earlier through its role in degrading the ER membrane protein INSIG1 [36]. With the new findings, an elaborate pathway was proposed in which UBXD8-p97-dependent degradation of INSIG1 enables the release of the SREBP1 transcription factor from the ER membrane, leading to its nuclear translocation, activation, and subsequent increase in SCD1 expression. SCD1 is a stearoyl-CoA desaturase that converts saturated fatty acids into monounsaturated fatty acids. The end result is lipid desaturation and maintenance of fluid cellular membranes.

Yet another interesting example of p97 influencing membrane dynamics relates to its role as a host protein in Zika virus infection [37]. The interaction between the virus NS4B gene and the host p97, along with p97 activity, induces mitochondria elongation and inhibits apoptosis. Inhibition of p97 under these conditions destabilizes viral replication factories, reverses the mitochondrial elongation phenotype, and increases the rate of apoptosis. These findings suggest that p97 inhibition may be an effective antiviral treatment for Zika virus, for which there are no available therapies to date.

## p97 and mitophagy regulation

**Figure 4:**
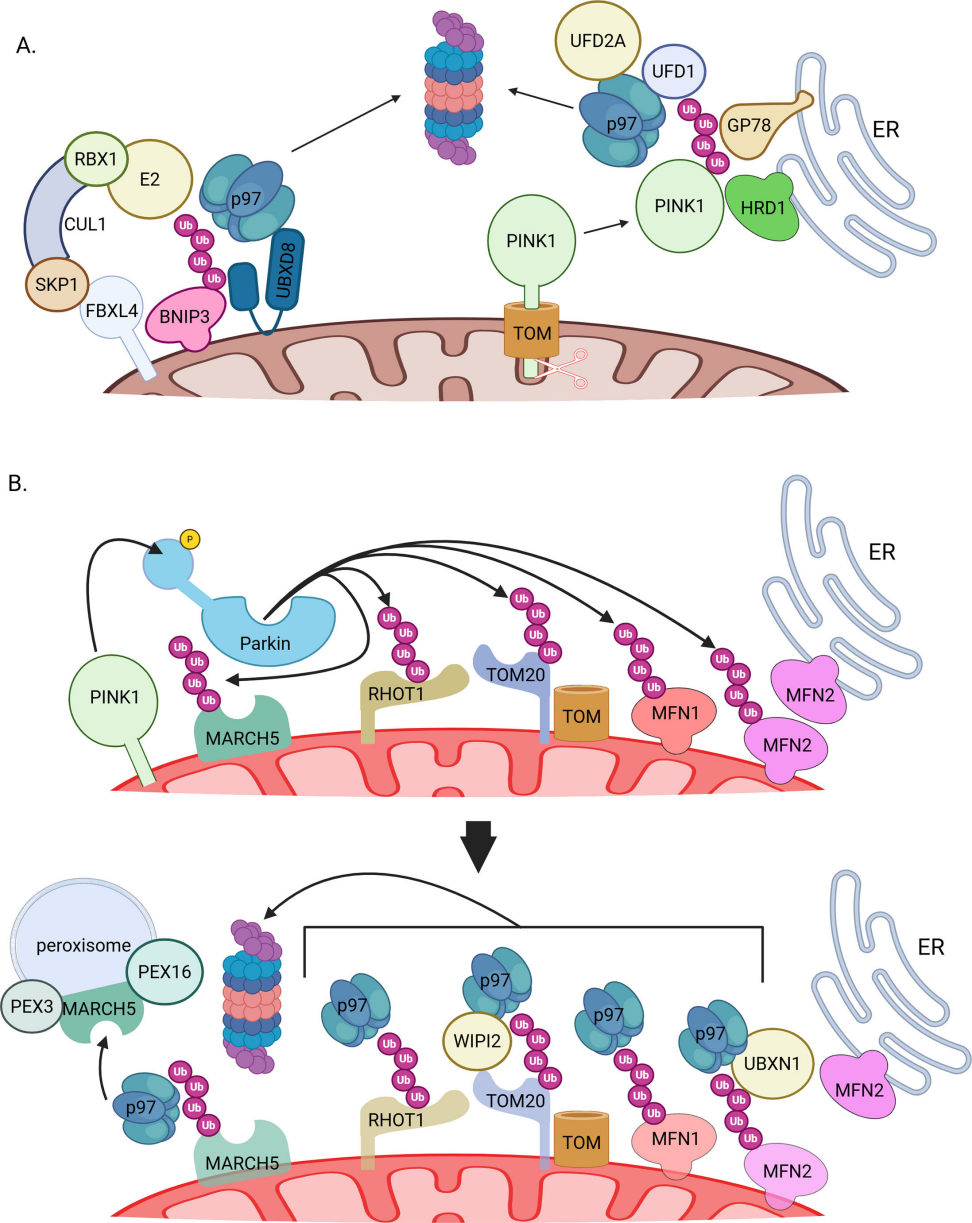
p97 in mitophagy regulation. **(A**) p97 in the prevention of basal mitophagy. Under basal conditions, the mitophagy receptor BNIP3 is ubiquitinated by SCF-FBXL4 and extracted by p97, while cleaved PINK1 is ubiquitinated by ER membrane-residing E3 ligases. Both substrates are degraded by proteasomes, thus preventing basal ubiquitin-independent and ubiquitin-dependent mitophagy, respectively. (**B**) p97 in PINK1-Parkin-dependent mitophagy. Following mitochondrial membrane depolarization, PINK1 phosphorylates and activates Parkin. OMM ubiquitination substrates of Parkin are extracted by p97 and its cofactors and are either degraded in proteasomes (MFN1/2, TOM20, RHOT1) or redistributed to peroxisomes (MARCH5). This step limits protein import and mitochondrial fusion, separating mitochondria from the ER to perpetuate mitophagy while preserving MARCH5 protein levels. Created in BioRender. Lab, G. (2025) https://BioRender.com/7b0vhbg.

Fission generates smaller, easier to engulf mitochondria—an essential preliminary step for mitophagy. The involvement of p97 in mitophagy was first described in relation to Parkin-dependent mitophagy [38]. Upon membrane depolarization, MFN1 and MFN2 are ubiquitinated by activated Parkin, leading to their degradation. Activation of Parkin happens through phosphorylation by the mitochondrial kinase PINK1, a typically short-lived protein that is converted to a stable form in the OMM, mainly after membrane depolarization. Under basal conditions, PINK1 is proteolytically cleaved in the IMS during its import into mitochondria, and a shorter fragment (52 kD) is released to the cytosol and degraded in the proteasome. The degradation of the 52 kD fragment depends on ERAD machinery, including the E3 ligases Hrd1 and GP78, the p97-Ufd1 complex, and the p97-associated Ub E4 ligase UFD2A [39] (Figure 4A).

p97 also directly regulates receptor-mediated/Parkin-independent mitophagy, a type of mitophagy that occurs via direct binding of autophagosomes to OMM-residing mitophagy receptors. UBXD8 and p97 facilitate the degradation of one of these mitophagy receptors, BNIP3, following its ubiquitination by the recently discovered OMM-residing E3 ligase SCF-FBXL4 [40]. The result is a decrease in basal mitophagy levels (Figure 4A). This study also demonstrated that the E3 ligase SCF‐FBXL4 associates with UBXD8‐p97.

In Parkin-overexpressing HeLa cells, p97 is recruited to depolarized mitochondria along with its cofactors Npl4, Ufd1, and p47 [41]. Knockdown of Npl4 or Ufd1 slows the degradation of MFN1 and MFN2 following mitochondrial depolarization. A separate study in HeLa cells overexpressing Parkin [42] identified UBXD1 as the recruitment factor of p97 to mitochondria upon depolarization (Figure 4B). Overexpression of UBXD1 increased mitophagy levels, while knockdown of UBXD1 had the opposite effect. The UBX domain of UBXD1 was necessary for its mitochondrial localization (Figure 2). In another study, also conducted in HeLa cells, UBXN1 was shown to recruit p97 to depolarized mitochondria [43] (Figure 4B). This recruitment depends on the UBX and UBA domains of UBXN1 (Figure 2) and aids mitophagy initiation by facilitating the removal of MFN2 from mitochondria. Overexpression of UBXD1 could rescue Parkin recruitment to depolarized mitochondria in UBXN1 KO cells, indicating a degree of redundancy (Figure 2) of these two UBX proteins. Expression of the dominant-negative QQ variant of p97 leads to the accumulation of MFN1, suggesting that p97 participates in MFN1 degradation (Figure 4B).

In *D. melanogaster*, VCP (p97) is recruited to neuronal mitochondria in a Parkin-dependent manner and is essential for proteasomal degradation of the mitofusin Marf [44] (Figure 3B). Additionally, the protein Clueless is important for Parkin-dependent Marf degradation, enabling mitochondria engulfment by autophagosomes [45]. Moreover, VCP overexpression leads to down-regulation of mitofusin levels in fly muscles and can rescue PINK1/Parkin mutant phenotypes [32]. A fly model of IBMPFD (inclusion body myopathy with early-onset Paget disease and FTD) expressing hyperactive VCP replicated disease phenotypes such as enhanced mitofusin degradation, fragmented mitochondria, and myopathy. Treatment with p97 inhibitors alleviated mitochondrial defects caused by this hyperactive mutant p97 found in IBMPFD patients [32].

In Huntington’s disease (HD) models, disease-related mutant Huntingtin (Htt) recruits p97 to mitochondria via direct protein-protein interactions [46]. Recruitment of p97 to mitochondria in this context leads to excessive mitophagy, causing a decrease in energy supply and overall neuronal survival. Mechanistically, mitochondrial-bound p97 recruits the autophagosome marker LC3B via two LC3-interacting regions, allowing for autophagosome recruitment and onset of mitophagy.

Another protein involved in the recruitment of VCP to depolarized mitochondria is WIPI2, an ATG protein associated with autophagy [47] (Figure 4B). In cells expressing Parkin, WIPI2 recruits p97 to depolarized mitochondria, enabling degradation of TOMM20 and promoting mitophagy. Chemical inhibition of p97 did not exacerbate the effect of WIPI2 deletion on TOMM20 clearance, suggesting they work together rather than in parallel pathways.

Another manner by which p97 promotes mitophagy is by dismantling MFN2 complexes at ERMCS, thus lowering ERMCS levels and gating subsequent steps of mitophagy (once again confirmed in HeLa cells overexpressing Parkin following CCCP treatment to depolarize mitochondria [33] (Figure 4B). In this context, p97 is recruited to depolarized mitochondria and lowers the levels of ubiquitinated MFN2 and MFN1. The authors suggested a model in which mitochondrial depolarization leads to rapid ubiquitination of MFNs, followed by p97-dependent extraction and ERMCS uncoupling. Detachment of mitochondria facilitated stable Parkin recruitment and ubiquitination of other OMM substrates, eventuating in mitophagy. In this context, an elegant *in-organelle* reconstitution of the system showed that MFN2 and VDAC1 ubiquitination are stimulated by p97. While MFN2 is also extracted by p97, VDAC1 is postulated to be too OMM-embedded to be extracted, due to its β-barrel channel structure.

More recently, the importance of p97-dependent MFN2 degradation for the progression of mitophagy has been called into question. A comprehensive proteomic study of iNeurons with endogenous Parkin levels showed no effect of p97 inhibition on mitophagy progression following depolarization [48]. Moreover, p97 inhibition only slowed the degradation of RHOT1 and MFN1 (Figure 4B) and had no significant effect on ubiquitination of other OMM Parkin substrates. Notably, phosphorylated Ub levels were also different from p97 inhibition in this model system, further contradicting the need for p97-dependent extraction of MFN2 for mitophagy progression in cells expressing endogenous Parkin.

A fascinating contribution of p97 to mitophagy involves the redistribution of select ubiquitinated substrates, rather than plain old extraction for the sake of degradation (Figure 1D). Following depolarization, activated Parkin ubiquitinates the OMM-resident E3 ligase MARCH5/MITOL on its residue K268 to set it up for extraction by p97 and redistribution to peroxisomes [49] (Figure 4B). The redistribution depends on the peroxins PEX3 and PEX16. By redistributing to peroxisomes, MARCH5 is able to evade lysosomal degradation along with damaged mitochondria.

## p97 in apoptosis regulation

**Figure 5:**
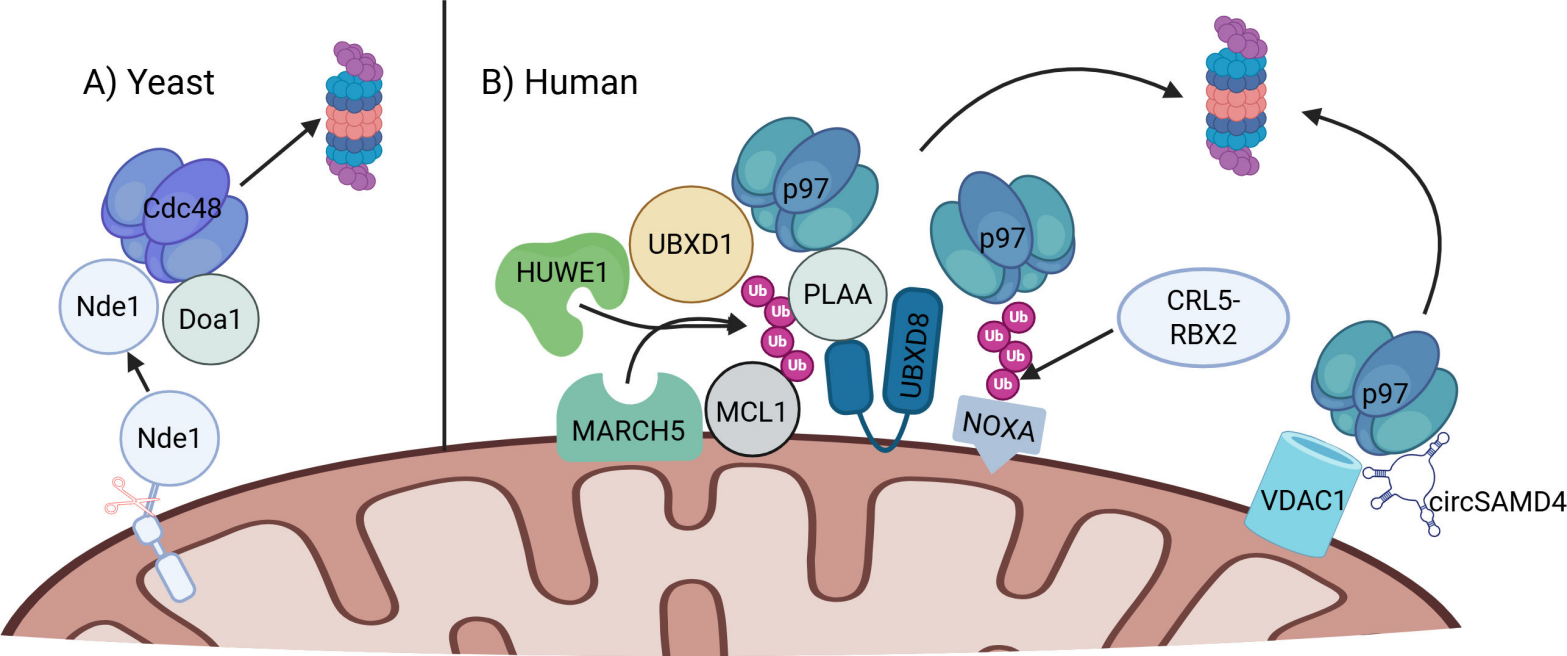
p97 in apoptosis regulation. (**A**) In yeast (*S. cerevisiae*), a cytosol-exposed topomer of Nde1 is cleaved in an unknown mechanism, and its cytosolic fragment is degraded by Cdc48-Doa and the proteasome, thereby preventing apoptosis. (**B**) In humans (*H. sapiens*), MCL1 is ubiquitinated by MARCH5 or HUWE1, extracted by p97 and its cofactors (PLAA, UBXD8, or UBXD1), and degraded in proteasomes. NOXA is ubiquitinated by CRL5 (SAG) and extracted by p97 and its cofactor UBXD8. VDAC1 is extracted by p97 and circSAMD4 and degraded in proteasomes. Created in BioRender. Lab, G. (2025) https://BioRender.com/g72i3e2.

The first report of mitochondrial involvement in Cdc48-regulated apoptosis came from studies of the Cdc48^S565G^ mutant yeast [50]. This strain showed increased levels of mitochondria-derived ROS, enlarged mitochondria, and increased cytochrome C release from mitochondria. A possible mechanism by which Cdc48 and its cofactor Doa1 participate in the regulation of apoptosis is through the degradation of a toxic cytosolic fragment of the NADH dehydrogenase (Nde1), associated with Complex I found in the IMM [51]. In respiration-deficient cells, this toxic fragment of Nde1 accumulates in the cytosol due to decreased import and increased release from mitochondria. In contrast, healthy cells degrade a small amount of this fragment through Cdc48-Doa1-dependent proteasomal degradation (Figure 5A). Cdc48 is not responsible for the extraction of Nde1 from the OMM. Still, it is important for the degradation of its soluble cytosolic fragment, hinting at the later-discovered role of Cdc48 in the degradation of tightly folded proteins rather than retro-translocation of mitochondrial proteins [3].

In mammalian cells, p97 was found to mediate the extraction of several short-lived apoptosis regulators from the OMM, allowing for their proteasomal degradation. One example is myeloid cell leukemia-1 (MCL1): it prevents apoptosis under basal conditions, but its rapid turnover allows cells to swiftly reduce its levels in response to stress, facilitating timely apoptotic responses. High constitutive expression ensures protection against accidental cell death, while rapid degradation provides regulatory flexibility [52]. In one study [53], p97 was shown to facilitate the retro-translocation of the anti-apoptotic protein MCL1 from isolated mitochondria by *in vitro* recapitulation. Involvement of p97 was dependent either on the OMM-residing E3 ligase MARCH5 or recruitment of the cytosolic HECT E3 ligase HUWE1/Mule for substrate ubiquitination [54,55]. Additionally, a separate study discovered that the mammalian homolog of the Ub-binding Cdc48-adaptor Doa1, PLAA (Figure 2), mediates the extraction and proteasomal degradation of MCL1 [56] (Figure 5B). The interaction between PLAA and MCL1 requires prior binding to p97 via the PLAA PUL domain (Figure 2). Other adapters may be involved too; in HD model cells, UBXD1 and p97 co-operate in the extraction of MCL1 from the OMM and its subsequent proteasomal degradation [57] (Figure 5B). Short-term proteasome inhibition stabilized MCL1, supporting its role in its turnover [58]. Separately, MCL1 degradation has recently been shown to depend also on the UBX protein UBXD8/FAF2, which recruits p97 to the OMM [13]. Overall, MCL1 degradation is aided by several p97 cofactors, implying redundancy in the regulation of this important anti-apoptotic factor. UBXD8 also plays a role in apoptosis by aiding in the p97-dependent degradation of the pro-apoptotic NOXA [13], which was previously shown to undergo ubiquitination by the CRL5-RBX2 E3 ligase to regulate apoptosis [59] (Figure 5B).

In cardiomyocytes, the circular RNA (circRNA) Samd4 was recently found to reduce oxidative stress, induce proliferation, and reduce apoptosis rates [60]. Mechanistically, circSamd4 binds p97 and recruits it to mitochondria, where together they down-regulate VDAC at the protein level (Figure 5B), thereby reducing the opening rate of the mitochondrial permeability transition pore (mPTP), an early step in apoptosis. This is the first report of a circRNA partnership with p97, paving a new avenue in p97 research.

The ALS-related p97^R191Q^ mutation was introduced in a neuroblastoma cell line to study the mechanistic aspects of disease progression [61]. Cells expressing p97^R191Q^ contain enlarged mitochondria and depolarized membrane potential. Mitochondria in these cells had increased oxygen consumption and calcium-induced mPTP opening, causing the mitochondrial depolarization phenotype. A recent structural study revealed that the R191Q single-site substitution disrupts the interaction between the N-D1 domains of p97, affecting the ADP-bound state of p97, rendering it hyperactive [62].

Taking together all the above observations, Cdc48/p97 appears to play a tightly regulated anti-apoptotic role across evolution, as mutations increase apoptosis rates.

## Future directions/open questions

As a ‘segregase’ or an ‘extractase’, one of the most obvious ways p97/VCP/Cdc48 influences mitochondria is by extracting proteins from the OMM and relaying them to the proteasome for degradation. It is important to note that not all p97 substrates listed in this review (Table 1) were directly proven to be extracted from mitochondria in a p97-dependent manner. Many show accumulation or decreased clearance following either p97 inhibition or down-regulation. Similarly, some accumulate upon perturbation of other steps in their degradation pathway (E3 ligase deletion, p97-adaptor deletion, proteasome inhibition), pointing to a p97 requirement. Studies seldom examine an entire process, often focusing on one or several steps of the pathway. To study and fully characterize a MAD process, one would have to find an E3 ligase, Ub-chain type, p97 cofactors involved, and the fate of the extracted substrate, as well as the cause and the outcome of this MAD.

**Table 1:** A list of mitochondrial p97/Cdc48 substrates

Protein substrate	Organism	Pathway	Proteins involved
Fzo1	Yeast, nematode	Membrane dynamics	Mdm30 [17], Vms1 [17], Doa1 [23], Ubx2 [25],
Mdm34, Msp1, Tom70	Yeast	Proteostatic MAD	Doa1 [23], Ufd1 [23], Npl4 [23], Rsp5 [23]
Sam35^ts^	Yeast	Quality control MAD	Ubx2 [25], Doa1 [25], San1 [25], Ssa1 [25], Sis1 [25], Npl4 [25], Ufd1 [25]
Sen2^ts^	Yeast	Quality control MAD	Ubx2 [25], Doa1 [25], Ubr1 [25], Ssa1 [25], Sis1 [25], Npl4 [25], Ufd1 [25]
MCL1^1^ [53]	Human, Mouse	Apoptosis	March5 [54], UBXD8 [13], UBXD1 [57], HUWE1 [55], PLAA [56]
MID49	Human	Mitochondrial fission	Ubxd8 [13], UBXD2 [13], MARCH5 [35,54]
MFN1	Human, Mouse	Mitophagy, Membrane dynamics	Parkin [44], Npl4 [41], Ufd1 [41], p47 [41], MGRN1 [34]
Marf	Fly	Mitophagy, membrane dynamics	Clueless [45]*, Parkin* [32]*, Mul1* [32]
MFN2^1^ [33]	Human	Mitophagy, Membrane dynamics	Parkin [33], UBXN1 [43]
RHOT1	Human	Mitophagy	Parkin [48]
TOM20	Human	Mitophagy	Parkin [47], WIPI2 [47]
BNIP3	Human	Mitophagy	UBXD8 [13], CUL1 [40], FBXL4 [40]
NOXA	Human	Apoptosis	UBXD8 [13], CUL5 [59]
Mdj1	Yeast	mitoTAD	Rsp5 [29], Ubx2 [28], Ufd1, Npl4
Sod2	Yeast	mitoTAD	Ubx2 [28]
Rip1	Yeast	mitoTAD	Ubx2 [28]
Kgd1, Pim1p	Yeast	Stress-induced MAD	Doa1 [27]
Nde1	Yeast	Apoptosis	Doa1 [51]
Vdac1	Mouse	Apoptosis	circSamd4^2^ [60]
TOM20	Thaliana	Quality control MAD	TTOP [24]
MARCH5/MITOL	Human	Mitophagy	Parkin [49], PEX3 [49], PEX16 [49]
PINK1	Human	Mitophagy	HRD1 [39], GP78 [39], UFD2A [39], UFD1 [39]

1 Directly shown to be extracted from mitochondria in a p97/Cdc48-dependent manner

2 Circular RNA

How does p97 recognize its mitochondrial substrates? The ubiquitination landscape on mitochondria under basal conditions is poorly characterized, but all Ub linkage types have been identified at mitochondria, with K29, K48, and K63 ubiquitination significantly increasing following proteasome inhibition [58]. This feature suggests that the proteasome is involved in continuously shaping the mitochondrial proteome. Independently, K6, K11, K48, and K63-linked Ub chains accumulate at mitochondria following membrane depolarization in Parkin-expressing cells [63]. Whether p97 recognizes its mitochondrial substrates directly, via recognition of a single Ub moiety, or by recognizing specific Ub-chain types remains unknown. A recent study found that branched K48-K63-branched Ub chains act as p97 recruitment signals [64], but no evidence for a role in mitochondria has yet been uncovered.

Is p97 involved in the degradation of nascent mitochondrial proteins? Mitochondrial stress can cause the accumulation and aggregation of nascent mitochondrial proteins, and in such cases, p97 is perhaps required for their efficient degradation. Under basal and stress conditions, some nascent mitochondrial proteins never reach the mitochondria and are degraded by cytosolic proteasomes. These proteins are likely chaperone-bound and kept in an unfolded state, hinting that p97 may not be involved in their degradation under basal conditions.

In some cases, the extraction of proteins from the OMM or from TOM complexes does not require p97 but is instead facilitated by a different AAA+ATPase, named ATAD1 (Msp1 in yeast) [65–67]. The interplay between these two ATPases, particularly in terms of substrate specificity, Ub dependence, and modes of activation, is poorly understood.

With the new gene-editing options enabled by CRISPR-Cas9 technology, the effects of pathological point mutations in p97 can be studied in cell culture, allowing for a deeper understanding of disease mechanisms. Moreover, mutations affecting p97’s interaction with specific adaptors, which play roles in mitochondrial regulation, could help discriminate between mitochondria-related and unrelated roles of p97.

To conclude, the role of p97 in mitochondrial biology is an area that warrants further investigation. New discoveries could open avenues for treating cancer and neurodegeneration, broadening our arsenal of drugs and thereby improving human health and quality of life.

**Summary points**

- P97 extracts proteins from the outer mitochondrial membrane, mainly for degradation by the proteasome.
- P97 regulates mitochondrial membrane fusion, mitophagy, and apoptosis through the degradation of specific mitochondrial proteins.
- Designated adaptors recruit p97 to ubiquitinated substrates on the mitochondria.

## Open Access

This article has been published open access under our Subscribe to Open programme, made possible through the support of our subscribing institutions, learn more here: https://portlandpress.com/pages/open_access_options_and_prices#conditional

## Abbreviations

ALS: amyotrophic lateral sclerosis
ATAD1: ATPase family AAA domain-containing protein 1
ATG: Autophagy-Related protein
CCCP: Carbonyl cyanide m-chlorophenylhydrazone
CRL5: Cullin RING ligase enzyme 5
Doa1: degradation of alpha2 1
ERAD: endoplasmic reticulum-associated degradation
ERMCS: endoplasmic reticulum mitochondria contact site
FAF2: Fas-associated factor 2
FTD: frontotemporal dementia
HD: Huntington’s disease
HEK293: Human Embryonic Kidney 293 cells
IMM: inner mitochondrial membrane
IMS: intermembrane space
MAD: mitochondria associated degradation
MCL1: myeloid cell leukemia-1
MFN1: Mitofusin 1
MFN2: Mitofusin 2
MID49: Mitochondrial dynamics protein 49
Mdm34: Mitochondrial Distribution and Morphology protein 34
NOXA: (Latin for damage); a pro-apoptotic member of the Bcl-2 protein family officially named PMAIP1, for Phorbol-12-myristate-13-acetate-induced protein 1
NPL4: Nuclear protein localization protein 4
OMA1: overlapping proteolytic activity with m-AAA protease 1
OMM: outer mitochondrial membrane
PEX3: Peroxisomal Biogenesis Factor 3
PEX16: Peroxisomal Biogenesis Factor 16
PFU: PLAA family ubiquitin-binding domain
PIM: Proviral Integration site for Moloney murine leukemia virus motif
PINK1: PTEN-induced putative kinase 1
PLAA: phospholipase A-2-activating protein
PUL: PLAP, Ufd3, and Lub1 domain
RBX2: RING-box protein 2
RHOT1: Mitochondrial Rho GTPase 1, A.K.A. Miro1
TOM20: Translocase of Outer Mitochondrial Membrane 20
TOM70: Translocase of Outer Mitochondrial Membrane 70
TOM: translocase of the outer membrane
UBA: ubiquitin-associated domain
UBX: ubiquitin regulatory X domain
UBXD8: ubiquitin regulatory X domain-containing protein 8
UBXN1: UBX Domain-Containing Protein 1
UFD1: Fusion Degradation protein
UFD2A: Ubiquitin fusion degradation protein 2a, A.K.A. UBE4B
UPS: ubiquitin proteasome system
Ub: ubiquitin
VCP: valosin-containing protein
VDAC: voltage-dependent anion channel
WIPI2: WD repeat domain phosphoinositide-interacting protein 2
circRNA: circular RNA
mPTP: mitochondrial permeability transition pore
mitoTAD: mitochondrial protein translocation-associated degradation
